# Application of 3D Animation Cluster System Based on Artificial Intelligence and Machine Learning

**DOI:** 10.1155/2022/2904607

**Published:** 2022-06-26

**Authors:** You Lv

**Affiliations:** Department of Fine Arts, Taiyuan Normal University, JinZhong, China

## Abstract

In many living phenomena, the behavior of social animals (such as ants, fish stocks, and birds) has attracted great attention, and many theories and models have emerged to simulate the behavior of biological communities. These studies are important in theory and practice and have wide application potential in optimization methods and product design, which leads to the so-called cluster intelligence. Development of the 3D animation system can be closely combined with cluster intelligence, and the full application of cluster intelligence is conducive to continuously improve the stability of animation system design, for animation system design to bring continuous design inspiration. The purpose of the development platform is to promote the development of enterprise projects, record each stage of the animation process in 3D, form a complete industrial chain, ensure the traceability and resilience of the whole process, and provide a comprehensive and effective solution for 3D animation production.

## 1. Introduction

With the development of life science, the research on the nature and phenomenon of life is not only limited to biology, but also extended to multidisciplinary fields such as mathematics and physics [1]. With the rapid development of information and communication technology, researchers began to develop mathematical models and simulate and analyze various life forms. Finally, a large number of artificial biological behavior systems were established on the basis of various life forms [2]. In recent years, many phenomena in life, such as the group behavior of social animals such as ants, fish stocks, and birds, have attracted great attention, and many theories and models have emerged to simulate the behavior of biological communities. This is the most important mechanism of collective intelligence and self-organization [3]. A study of collective intelligence self-organization helps us to design methods to solve distribution problems and 3D animation systems [4]. In order to better apply collective wisdom theory to the 3D animation system, it is necessary to use individual and collective intelligence system patterns to study cluster stability and intelligent behavior mechanisms [5]. Theories related to collective wisdom and applied technology are important in theory and practice and have wide application potential in optimizing methods and designing distributed intelligent systems.

During the 3D animation production process, many business data and media resource files are produced, which are the pillars of enterprise development, and sound and effective regulatory data are the cornerstone of enterprise development [6]. The current method of corporate data storage takes different forms. By analyzing its own operation reasonably and selecting appropriate storage, it can lay a good foundation for the development of the repository [7]. The proliferation of formal and semiregular data makes information storage an obstacle to development. In the 3D animation industry, the choice of appropriate cloud storage services has become an option for enterprises to obtain data at anytime and anywhere, saving local storage costs, regardless of maintenance matters, the lack of personnel in the production process due to data confusion, the speed of the industry is limited, the ratio between staff and tasks is very low, it is difficult for system managers to obtain information project progress, and the distribution of staff and workers only partially understand the tasks they undertake and the tasks they accomplish [8]. The purpose of the development platform is to promote the development of enterprise projects, to record each stage of the animation process in 3D, to form a complete industrial chain, to achieve traceability and resilience of the whole process, and to provide a comprehensive solution, and also in 3D animation production efficiency [9].

## 2. Related Work

On cluster intelligence research, the literature proposed cluster intelligence models to solve such problems, known as AER models, which introduce the concept of collective intelligence into traditional problems of traditional artificial intelligence and solve problems of N queen, dyeing problems [10]. The literature has done a lot of work in analyzing the salient features of space systems. The report states that group behavior is revealed only when the number of individuals exceeds a certain value [11]. The evolution neural network is used to control the robot's behavior. and the influence of robot parameters is analyzed. The literature finally concludes that the system is at the edge of confusion when the panel has the highest success rate within the mission area. The mission made an important assumption that changes, or connections might be more inclined to the venue in the course of evolution; that is, the resulting changes were not entirely random and were often more likely to be mutated [12].

As for the design of the animation system, with the development of related cultural industries in China, the demand for 3D media animation products is increasing, thus developing cooperative management software for many animation products [13]. Further development of animation industry at home and abroad, its characteristic is to fully apply animation production software. The literature shows that unlike the way hard disks store data, cloud storage is an area of interest to all because of its unique advantages, and cloud storage capacity can be expanded at any time as user needs change and income levels change [14]. As noted the literature, cloud storage is Internet-based and contains vast amounts of information about storage, which is a simple extension capability for unified data management to facilitate resource sharing [15]. The system allows to connect animation production, and the literature shows that due to the lack of technical information and the literature, the actual development process takes more time, which enables the transfer of resources as the process progresses and promotes development and production [16]. At the same time, due to the understanding of industry in a specific development process, there is no complete control program found in the whole animation production process. This has produced a lot of back and forth, the literature pointed out that blue sea creative cloud, which has rich experience in animation production, works in five main areas, namely, project portfolio, initial incubator, online collaboration, and promotion of farms and media, and it can be seen as a new development prospect as domestic industry [17].

## 3. Stability Analysis of Cluster Intelligent System Based on Individual Model

### 3.1. Group Self-Aggregation Analysis in Complex Dynamic Environment

In order to facilitate the analysis, a simple condensation system is proposed to analyze the self-aggregation of the population, which can provide a motion control equation for everyone:

(1) $$\begin{array}{c} {\overset{•}{x}}_{i}=v_{i},\\ m_{i}{\overset{•}{v}}_{i}=u_{i}. \end{array}$$

All individuals within *i* observable range are centered:

(2) $$\begin{array}{c} {\overset{-}{x}}_{0}^{i}=\frac{1}{N_{i}}\underset{j\in S_{i}}{\sum }x_{j}. \end{array}$$

Convergence requires interaction between the organizations of the joint system, and the simplest way to ensure that they can be clustered is to introduce interindividual gravity. Similarly, in order to avoid collision with others, when they approach a certain distance, they must keep a distance from the other party, so it is necessary to cross-examine between the two parties. Linear gravity and penetration are defined as

(3) $$\begin{array}{c} f_{a}\left(x,y\right)=\left\{\begin{array}{cc} -a\left(x-y\right), & \rho \left(x,y\right)<r\\ 0, & \rho \left(x,y\right)\geq r \end{array}\right\},\\ f_{r}\left(x,y\right)=\left\{\begin{array}{cc} b\text{exp}\left(\frac{-{\left\|x-y\right\|}^{2}}{c}\right)\left(x-y\right), & \rho \left(x,y\right)<r\\ 0, & \rho \left(x,y\right)\geq r \end{array}\right\}. \end{array}$$

**Figure 1:**
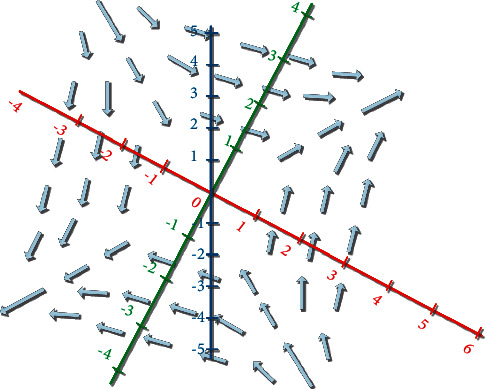
Gradient field.

According to the environmental profile information, the gradient information of the environmental change at the location *y* can be obtained. Gradient field is shown in Figure 1:


(4) $$\begin{array}{c} \nabla_{y}\sigma \left(y\right)=\frac{A}{l}\text{exp}\left(-\frac{{\left\|y-c_{\sigma }\right\|}^{2}}{l}\right)\left(y-c_{\sigma }\right). \end{array}$$

The movement of an individual is directed towards the negative gradient force of the gravitational field, the repulsion field, and the environmental field, so there is

(5) $$\begin{array}{c} \tilde{u_{i}}=-\nabla U_{i}=\underset{j\in S_{i}}{\sum }\left[f_{a}\left(x_{i},x_{j}\right)\right]-\nabla_{xi}\sigma \left(x_{i}\right). \end{array}$$

To sum up, it is possible to

(6) $$\begin{array}{c} \overset{•}{x_{i}}=\underset{j\in S_{i}}{\sum }\left[f_{a}\left(x_{i},x_{j}\right)+f_{r}\left(x_{i},x_{j}\right)\right]-\nabla_{xi}\sigma \left(x_{i}\right),\\ =\underset{j\in S_{i}}{\sum }\left[-a+b\text{exp}\left(-\frac{{\left\|x_{i}-x_{j}\right\|}^{2}}{c}\right)\right]\left(x_{i}-x_{j}\right)-\nabla_{xi}\sigma \left(x_{i}\right). \end{array}$$

Assuming that the position of the target point is constant, the time derivative can be obtained:

(7) $$\begin{array}{c} {\overset{•}{V}}_{\sigma }^{i}={\overset{•}{e}}_{\sigma }^{i}Te_{\sigma }^{i}={\overset{\_•}{x}}_{0}^{i}Te_{\sigma }^{i},\\ ={\left[-\frac{1}{N}\cdot \frac{A}{l}\underset{j\in S_{i}}{\sum }\text{exp}\left(-\frac{{\left\|x_{j}-c_{\sigma }\right\|}^{2}}{l}\right)\left(x_{j}-c_{\sigma }\right)\right]}^{T}e_{\sigma }^{i}. \end{array}$$

When the individual *i* observation center is above the *R* position, the center position moves to the center of the destination until the vector area is R. At this time, everyone is in their respective observation area, and the whole group is close to the target site.

Information about a single environmental gradient is biased. Formula (4) should read:

(8) $$\begin{array}{c} \nabla_{y}\overset{\wedge }{\sigma }\left(y\right)=\frac{A}{l}\text{exp}\left(-\frac{{\left\|y-c_{\sigma }-d_{\sigma }^{i}\right\|}^{2}}{l}\right)\left(y-c_{\sigma }-d_{\sigma }^{i}\right). \end{array}$$

In a dynamic and quiet environment, the movement of the target in the system reaches the following limit: the target direction changes randomly, but the speed does not exceed 1. The experimental results show that although individuals were initially dispersed, they were increasingly close to the target. The Group's objectives and course of action demonstrate that wherever it arrives, the Group has reason to operate in the Joint Control Area Centre. when the target position is constant, more chaotic shifts are generated, which is mainly due to the stable buffering effect in the previous theorem.

With local perception, individuals gather together through gravity and can move. Attraction is used to balance the rise of gravity, so that a group of people do not get too close, and information about environmental gradients enables the group to move to a target and gather in the field. Individuals are able to take advantage of changes in time and environment, which in turn leads to effective accumulation. Experimental results show that the increase of gravity between people reduces the scope of group convergence, speeds up its speed, and strengthens the impact of partnerships. Enhanced tensile strength increases the intersection of groups and reduces their opportunities for cooperation. In low-noise environments, people in groups can easily identify other individuals and, ultimately, converge targets in a particular community in the region. However, depending on the intensity of the noise, the final distribution patterns of individuals within the community may also change, which are formed according to the external characteristics of the organization. The target group can adapt to the change of environment quickly and enter the target area, which indicates that the interaction between individual and environment in the collective intelligence system can improve the adaptability of the group.

### 3.2. Individual Model of Ring Classification and Stability Analysis

The motion of each object can be regarded as individual motion. Accordingly, according to the previous assumptions, the *i* of objects in type I have the following equations of motion in the course of being transported:

(9) $$\begin{array}{c} {\overset{•}{x}}_{1}^{i}=\overset{m_{1}}{\underset{j=1,j\notin i}{\sum }}g_{1}\left(x_{1}^{i}-x_{1}^{j}\right),\\ i=1,\ldots ,m_{1}. \end{array}$$

Here are the following forms:

(10) $$\begin{array}{c} g_{1}\left(y\right)=-y\left[g_{a}^{1}\left(\left\|y\right\|\right)-gr_{a}^{1}\left(\left\|y\right\|\right)\right]\\ =-y\left[a_{1}-b_{1}\text{exp}\left(-\frac{{\left\|y\right\|}^{2}}{c_{1}}\right)\right]. \end{array}$$

There will be the following equations of motion during handling:

(11) $$\begin{array}{c} {\overset{•}{x}}_{2}^{i}=\overset{m_{2}}{\underset{j=1,j\neq 1}{\sum }}g_{2}\left(x_{2}^{i}-x_{2}^{j}\right)+\overset{m_{1}}{\underset{j=1}{\sum }}g_{12}\left(x_{2}^{i}-x_{2}^{j}\right),\\ i=1,\ldots ,m_{2}. \end{array}$$

The terms of gravity and repulsion of type I objects to type II objects are defined as follows:

(12) $$\begin{array}{c} g_{2}\left(y\right)=-y\left[g_{a}^{2}\left(\left\|y\right\|\right)-g_{r}^{2}\left(\left\|y\right\|\right)\right]\\ =-y\left[a_{2}-b_{2}\text{exp}\left(-\frac{{\left\|y\right\|}^{2}}{c_{2}}\right)\right], \end{array}$$

(13) $$\begin{array}{c} g_{12}\left(y\right)=-y\left[g_{a}^{12}\left(\left\|y\right\|\right)-g_{r}^{12}\left(\left\|y\right\|\right)\right]\\ =-y\left[a_{12}-b_{12}\text{exp}\left(-\frac{{\left\|y\right\|}^{2}}{c_{12}}\right)\right]. \end{array}$$

According to formula (11),

(14) $$\begin{array}{c} {\overset{\_•}{x}}_{2}=-\frac{1}{m_{2}}\overset{m_{2}}{\underset{i=1}{\sum }}\overset{m_{2}}{\underset{j=1,j\neq 1}{\sum }}g_{2}\left(x_{2}^{i}-x_{2}^{j}\right)-\frac{1}{m_{2}}\overset{m_{2}}{\underset{i=1}{\sum }}\overset{m_{2}}{\underset{j=1}{\sum }}g_{12}\left(x_{2}^{i}-x_{1}^{j}\right)\\ =-\frac{1}{m_{2}}\overset{m_{2}}{\underset{i=1}{\sum }}\overset{m_{2}}{\underset{j=1}{\sum }}\left[g_{a}^{12}\left(\left\|x_{2}^{i}-x_{1}^{j}\right\|\right)-g_{r}^{12}\left(\left\|x_{2}^{i}-x_{1}^{j}\right\|\right)\right]\left(x_{2}^{i}-x_{1}^{j}\right). \end{array}$$

Formula (13) is replaced by formula (14):

(15) $$\begin{array}{c} {\overset{\_•}{x}}_{2}=-\frac{1}{m_{2}}\overset{m_{2}}{\underset{i=1}{\sum }}\overset{m_{1}}{\underset{j=1}{\sum }}a_{12}\left(x_{2}^{i}-x_{2}^{j}\right)+\frac{1}{m_{2}}\overset{m_{2}}{\underset{i=1}{\sum }}\overset{m_{1}}{\underset{j=1}{\sum }}g_{r}^{12}\left(\left\|x_{2}^{i}-x_{1}^{j}\right\|\right)\left(x_{2}^{i}-x_{1}^{j}\right), \end{array}$$

of which

(16) $$\begin{array}{c} \overset{m_{2}}{\underset{i=1}{\sum }}\overset{m_{1}}{\underset{j=1}{\sum }}a_{12}\left(x_{2}^{i}-x_{2}^{j}\right)=a_{12}\overset{m_{2}}{\underset{i=1}{\sum }}\overset{m_{1}}{\underset{j=1}{\sum }}\left(x_{2}^{i}-x_{1}^{j}\right)\\ =a_{12}\overset{m_{2}}{\underset{i=1}{\sum }}\left(m_{1}x_{2}^{i}-m_{1}{\bar{x}}_{1}\right)\\ =a_{12}m_{1}m_{2}\left({\bar{x}}_{1}-{\bar{x}}_{2}\right). \end{array}$$

Collection

(17) $$\begin{array}{c} V_{12}=\frac{1}{2}{\left\|e_{12}\right\|}^{2}=\frac{1}{2}e_{12}^{T}e_{12}. \end{array}$$

For the Lyapunov function of the system, V of calculation_12_, the time derivative can be obtained:

(18) $$\begin{array}{c} {\overset{•}{V}}_{12}={\left({\overset{\_•}{x}}_{2}-{\overset{\_•}{x}}_{1}\right)}^{T}e_{12}\\ ={\overset{\_•}{x}}_{2}^{T}e_{12}\\ =-m_{1}a_{12}{\left\|e_{12}\right\|}^{2}\\ +\frac{1}{m_{2}}\overset{m_{2}}{\underset{i=1}{\sum }}\overset{m_{1}}{\underset{j=1}{\sum }}g_{r}^{12}\left(\left\|x_{2}^{i}-x_{1}^{i}\right\|\right){\left(x_{2}^{i}-x_{1}^{i}\right)}^{T}e_{12}\leq -m_{1}a_{12}{\left\|e_{12}\right\|}^{2}+\frac{1}{m_{2}}\overset{m_{2}}{\underset{i=1}{\sum }}\overset{m_{1}}{\underset{j=1}{\sum }}g_{r}^{12}\left(\left\|x_{2}^{i}-x_{1}^{i}\right\|\right){\left(x_{2}^{i}-x_{1}^{i}\right)}^{T}\left\|e_{12}\right\|\leq -m_{1}a_{12}\left\|e_{12}\right\|\left(\left\|e_{12}\right\|-\frac{1}{a_{12}m_{1}m_{2}}\overset{m_{2}}{\underset{i=1}{\sum }}\overset{m_{1}}{\underset{j=1}{\sum }}g_{r}^{12}\left(\left\|x_{2}^{i}-x_{1}^{i}\right\|\right)\left\|x_{2}^{i}-x_{1}^{i}\right\|\right). \end{array}$$

Since the repulsion in formula (13) is bounded, it can be obtained:

(19) $$\begin{array}{c} g_{r}^{12}\left(\left\|x_{2}^{i}-x_{1}^{j}\right\|\right)\left\|x_{2}^{i}-x_{1}^{j}\right\|\leq b_{12}. \end{array}$$

(1) Available:
  (20) $$\begin{array}{c} \left\|e_{12}\right\|\leq \frac{1}{a_{12}m_{1}m_{2}}\overset{m_{2}}{\underset{i=1}{\sum }}\overset{m_{1}}{\underset{j=1}{\sum }}g_{r}^{12}\left(\left\|x_{2}^{i}-x_{1}^{j}\right\|\right)\left\|x_{2}^{i}-x_{1}^{j}\right\|\leq \frac{b_{12}}{a_{12}}\overset{\Delta }{=}\varepsilon_{12}. \end{array}$$
(2) Selection

(21) $$\begin{array}{c} V_{2}^{i}=\frac{1}{2}{\left\|e_{2}^{i}\right\|}^{2}\\ =\frac{1}{2}e_{2}^{i^{T}}e_{2}^{i},\\ {\overset{•}{V}}_{2}^{i}={\left({\overset{•}{x}}_{2}^{i}-{\overset{\_•}{x}}_{1}\right)}^{T}e_{2}^{i}={\overset{•}{x}}_{2}^{i}Te_{2}^{i}\\ =-\overset{m_{2}}{\underset{j=1}{\sum }}a_{2}{\left(x_{2}^{i}-x_{2}^{j}\right)}^{T}e_{2}^{i}+\overset{m_{2}}{\underset{j=1,j\neq i}{\sum }}g_{r}^{2}\left(\left\|x_{2}^{i}-x_{2}^{j}\right\|\right){\left(x_{2}^{i}-x_{2}^{j}\right)}^{T}e_{2}^{i}-\overset{m_{2}}{\underset{j=1}{\sum }}a_{2}{\left(x_{2}^{i}-x_{1}^{j}\right)}^{T}e_{2}^{i}\\ +\overset{m_{2}}{\underset{j=1,j\neq i}{\sum }}g_{r}^{2}\left(\left\|x_{2}^{i}-x_{1}^{j}\right\|\right){\left(x_{2}^{i}-x_{1}^{j}\right)}^{T}e_{2}^{i}\leq -a_{2}m_{2}\left(x_{2}^{i}-{\bar{x}}_{2}\right)e_{2}^{i}+m_{2}b_{2}e_{2}^{i}-a_{12}m_{1}{\left(x_{2}^{i}-{\bar{x}}_{2}\right)}^{T}e_{2}^{i}+m_{1}b_{12}e_{2}^{i}\\ \leq -a_{2}m_{2}\left(\left\|x_{2}^{i}-{\bar{x}}_{2}\right\|\right)\left\|e_{2}^{i}\right\|+m_{2}b_{2}\left\|e_{2}^{i}\right\|-a_{12}m_{1}{\left\|e_{2}^{j}\right\|}^{2}+m_{1}b_{12}\left\|e_{2}^{i}\right\|=a_{12}m_{1}\left\|e_{2}^{j}\right\|\left(\left\|e_{2}^{i}\right\|-\frac{a_{2}m_{2}}{a_{12}m_{1}}\left(\left\|x_{2}^{i}-{\bar{x}}_{2}\right\|\right)-\frac{b_{2}m_{2}}{a_{12}m_{1}}-\frac{b_{12}}{a_{12}}\right). \end{array}$$

According to previous studies, yes, there are $t\overset{⟶}{}\infty$:

(22) $$\begin{array}{c} \left\|x_{2}^{i}-{\bar{x}}_{2}\right\|\leq \frac{b_{2}}{a_{2}}. \end{array}$$

Available

(23) $$\begin{array}{c} \left\|e_{2}^{i}\right\|\leq \frac{a_{2}m_{2}}{a_{12}m_{1}}\left(\left\|x_{2}^{i}-{\bar{x}}_{2}\right\|\right)+\frac{m_{2}b_{2}}{a_{12}m_{1}}+\frac{b_{12}}{a_{12}}\leq \frac{2b_{2}m_{2}}{a_{12}m_{1}}+\frac{b_{12}}{a_{12}}\overset{\Delta }{=}\varepsilon_{2}. \end{array}$$

The increase of gravity between two types of objects will make the structure of the ring more compact. The resistance of the first type of object to the second type of object increases, and the resistance of the second type of object to the ring structure increases, which will increase the repulsion of space. The above analysis provides an analytical model for the classification of two types of objects, which can be extended to ring classification, and other types of classification.

### 3.3. Self-Organizing Factors of Cluster Intelligent System under Individual Model

The two different patterns in this chapter show that although the patterns of interaction between individuals are similar, there are final structures or patterns. Therefore, in the intelligence system of the group, the formation of different modes does not necessarily need to change the way of individual interaction but only needs to adjust the noise intensity of some parameters. The increase of gravity intensity can produce different forms of system behavior. The interaction between individuals affects the final results of the intelligence system, and the positive and negative factors of collective system feedback are balanced, affecting the performance of the system and the level of the system group self-service model. If the tensile strength changes, the affinity between individuals of a population may also vary. It can be said that feedback helps collaboration, and negative feedback hinders collaboration between groups to some extent. Therefore, the individual model introduces the basic elements of the organization system, converts the individual motion equation into the group-driven model-based dynamics 3D animation equation; theoretically, it allows to control the motion of animation groups over 3D. When the gravity and penetration balance of the model, 3D animation can be realized and controlled.

The analysis of a cluster-based single intelligent system model shows that the interaction between individuals not only affects the system but also affects the environment. In the field of intelligent control, the interaction between people and the hidden communication framework can be direct or indirect. However, regardless of the interactive approach, the rules governing individuals within groups are simple and uniform.

Self-regulation can be regarded as the basis of collective cooperation and the simplest prototype of the intelligent cluster algorithm. In data analysis, clusters can be based on the behavior of ants as “corpse clustering,” and a single model can be used. Finally, self-regulation and information on environmental gradients can be used as models to influence group location, and classification phenomena can produce expected results by enhancing interaction between different target groups.

## 4. Design of 3D Animation Cluster System Based on Artificial Intelligence and Machine Learning

**Figure 2:**
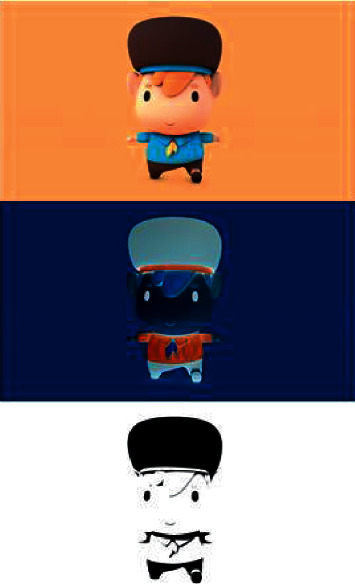
3D animation design.

In the process of system design, there should be certain system design objectives and system design principles, which is the root cause of system design as a whole. The system design principles and objectives should reflect differences and particularity, which must be generally applicable to most personnel management. For the system design of 3D animation products, we must reflect a high degree of flexibility, which should be reflected not only in the flexibility of task allocation, but also in the flexibility of personnel flow. Constantly enrich and develop the system to provide a solid foundation for animation product development. 3D animation design is shown in Figure 2.

### 4.1. System Design Objectives and Principles

The system should be designed to take into account its universality and its differences and specificities so that the whole system can form a single system.

First, the objectives of the system need to be clearly defined, which is the starting point of the design. The whole system needs to effectively integrate the storage, personnel management, capability control, and other units of media resources. All these should be supported by the media product production process, consistent with the business process, and complete the entire system of production products. The resource storage module provides the most basic requirements in the media production process and ensures that members of any group can quickly understand how to use the system.

Among them, personnel management, including mission management, is universal, and the users of the system have not yet adopted the new concept, but some adjustments have been made to the control unit of the Authority in the light of the specific characteristics of the Authority. The degree of control over rights varies from the project team to the core members of the project team. The purpose of the design is to take into account the differences in the production process, such as 3D animation products, and to allow system administrators to adjust according to the specific conditions they face in producing 3D animation products. Product specifically achieved consistency and diversity, and special attention should be paid to the flexibility of the system for review. The system is readily available to project managers to enable them to reallocate tasks and staff in line with the development of the project, increase flexibility in project support business processes, and facilitate staff mobility.

**Figure 3:**
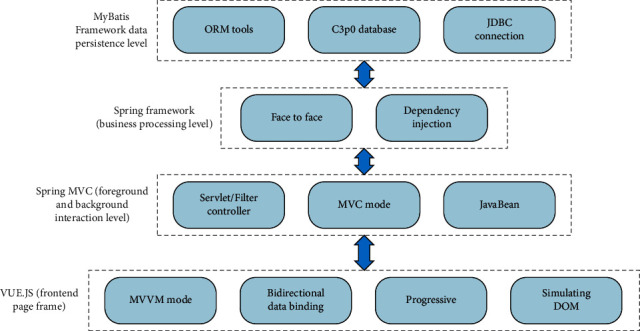
Technical framework of the system.

Finally, special attention should be paid to the expansion and transplantation of the system, in particular the standardization and rationalization of the interface provided through investment policy reviews. Using Tomcat JBOS in servers that support the current relational database (main database), the system can be installed, so that the system can fully support the development and production of 3D of animation products and establish a mature system that takes into account universality and difference. Technical framework of the system is shown in Figure 3 for details.

### 4.2. System Deployment Architecture Design

Subparagraphs (*A*) and (B) are at the core of the cloud storage platform and require the responsibility and interaction of the database to store the information needed to access the archives, as well as the core and interactive functions of the distributed storage system, which are installed in the Linux system.

**Figure 4:**
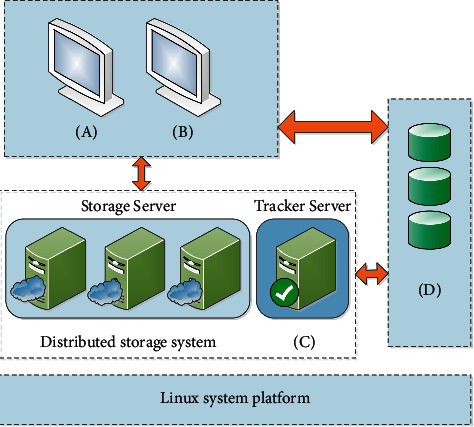
System deployment architecture diagram: (a) a secondary management service provided on behalf of a development system, (b) which provides interface services from outside the storage system distributed on the system platform, (c) which uses FASDF as distributed files; it is primarily responsible for the storage of animation resource files. (d) MySQL contains master file information, Mongodb.LaMysQL the database contains user information, rights information, group information, and mission information and mission-related changes.

Figure 4 shows the deployment structure of the system components.

**Figure 5:**
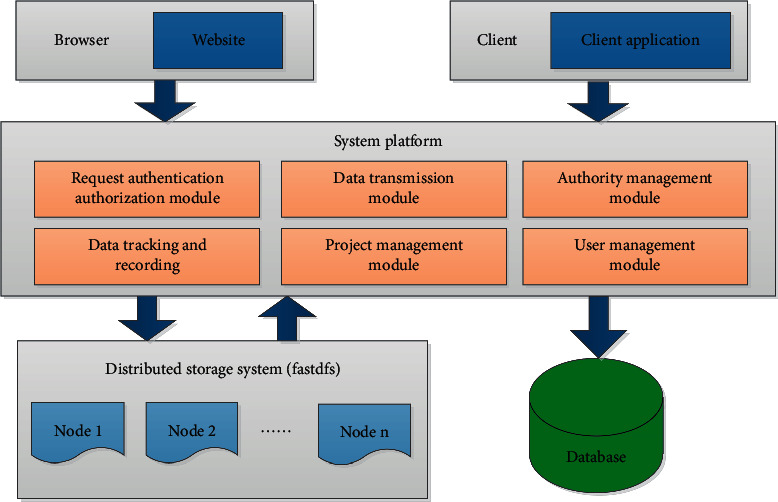
Logical structure of the system.

Figure 5 shows the logical structure of the system. When users use it, they first request verification module interaction, then complete the authentication of the license, and then continue to use other systems.

### 4.3. System Functional Architecture Design

The 3D animation production collaborative management system mainly includes data storage and access, data tracking and recording module, verification application module, personnel project management, work assignment control, and work analysis statistics.

The whole system platform can be divided into two parts, one of which is dedicated to system management, including project management, member management, group management, power management, and system statistical analysis. Part of the investment policy review service provided outside the system helps third-party access, including information on interfaces such as authentication, loading of animation files, downloading of drawings, access to historical files, and establishment of animation project structure directory.

### 4.4. System Testing

The following is an overall test of the model, reflecting the feasibility and challenge evidence of the system. The test contents include system recording, information function of system management, system project management, system staff management, control data of system authority, task assignment, analysis, and presentation unit, by testing each function in the function unit, the purpose is to cover all situations, so that all functions can be fully and detailed tested in Table 1, and the registration module must be tested before the system is used.

The test of the related functions of system animation project management is shown in Table 2.

The related functions of the system personnel management module are shown in Table 3.

The specific test of system rights management and storage functions is shown in Table 4.

The test of the related functions of the system task management module is shown in Table 5.

The test of the related functions of the data statistical analysis module of the system is shown in Table 6.

## 5. Conclusion

With the progress of science and technology and the rapid development of biology, people establish a large number of biological population models and theories through the observation of many populations. The model and theory of this biological group have gradually developed to produce cluster intelligence. Through the study of cluster intelligence, people further explore the nature of life, to deeply study the causes of various life phenomena, biology and physics, chemistry, and other disciplines are closely linked, promoting the integration of disciplines. At the same time, the study of life behavior provides reference for the development of modern society. Development of the 3D animation system can be closely combined with cluster intelligence, and the full application of cluster intelligence is conducive to continuously improve the stability of animation system design, for animation system design to bring continuous design inspiration.

Cluster wisdom originates from the characteristics of micro and unpredictable intelligent behavior displayed by individuals at the macro level through simple interaction. Individuals in the system can only realize complex behavior patterns and display collective wisdom through simple interaction. Compared with the interaction of other individuals and environments, local collective wisdom emphasizes the influence of “learning” on individual behavior, which adapts to the environment in collective intelligence systems by collecting and processing information. Each individual is not a separate substitute, but with the development of information technology, the demand for media products is greatly increased. This paper mainly puts forward a new method to adapt or solve many difficulties in producing a large number of animation resources products, because the production of these products requires a lot of time and energy. This method has not contributed much to the development of 3D national animation products, and it is expected that China will produce more high-quality animation products.

## Figures and Tables

**Table 1:** Login registration function test table.

Test function	Test operation	Test data	Test results
Mail delivery	Enter the address of the message to be sent, and click send	Address of receiving mail	Mail received successfully
System registration	Enter the registered enterprise name, password, confirmation password and verification code information related content, click registration	Testing enterprises	Successful registration, relevant data in the database
System login	Enter username and password click login	The test data are just registered information	Login successfully, enter the system

**Table 2:** Project module test table.

Test function	Test operation	Test data	Test results
Item query	Enter the keyword information for the query item and click on the query	Enter “Xin Qiji”	The results showed that all the projects related to Xin Qiji
Project creation	Click the Project Create button to fill in the project information	Project name “A”, project structure “A structure”	Project creation success
Project freeze	Select the item and click the freeze button	A “project	“A” projects frozen successfully
Project archiving	Select the item and click the archive button	A “project	“A” project archiving success
Project structure	Go to the project structure page, create a new directory hierarchy, and then complete the process steps with the project structure	No	Successful project structure
Project process definition	Go to the project process page, click the add process button, add steps according to the specific circumstances of the project	The steps in the added process are “model,” “light,”,” map,” “bind ,” composition, “animation”	Successful project process creation
Project process modification	Go to the project process page and select the process to be modified	Add the “special case” step	Successful project process modification

**Table 3:** Personnel management module test table.

Test function	Test operation	Test data	Test results
Increase in membership	Click the add person button, fill in the person information, click the finish button	Name + password	Personnel addition success
Membership enquiries	Enter the query data and click the query button	Input “Zhang”	Show the person associated with Zhang
Add groups	Click add group button, fill in group information, click finish button	Group Name “A Group” Group Profile	Add group success
Groups add members	Select the group, click the add button, and select the member to add	Choose Group “A”, Select Members”	Members were successfully added to groups
Group queries	Enter group page, enter group information, click query	Enter “A”	Check out all a project information

**Table 4:** Competence management and storage module test table.

Test function	Test operation	Test data	Test results
Personal authority	Freeze or activate personal authority	Zhang San	Cannot access the repository after freezing, can continue to access after activation
Group permissions	Freeze or excite group permissions	“A groups”	All members of the frozen group cannot be accessed after activation
Permission validation	userid and usersecrt access code obtained through URL request, (3) use code to get access accessToken to get access	userid = “599 d99e948df5e2223239065” usersscretuserid = ”ujliaHzfltcJg”	Successful code and access token accessToken, and access to relevant data
Resource upload	The upload of the resource is not completed by code verification, and the terminal continues to upload	Upload pictures	Upload success
Breakpoint continuation	Code run validation	Upload pictures	Upload success
Resources download	Test operation	Download Picture	Download success

**Table 5:** Test table for the task management module.

Test function	Test operation	Test data	Test results
Examination and verification	Go to the task page and select the task tab to be assigned, (2) select the task name, then you can view the content if (3) meet the criteria click pass otherwise click reject	Task name = “A model” through task	Success
Rejected tasks	(1) Go to the task page and select rejected task, (2) view the reason for the rejected task	Task name = “A Model”	Task loading changed to rejection
Create tasks	(1) Enter the task interface, click create task button, (2) pop up the dialog box about task creation to fill in the relevant content about task, (3) after filling in, click OK	Task name = “A Model”,	The task was created successfully and the information was fully consistent with the test data
Task redistribution	After (1) task creation is successful, click modify task, (2) reselect the task executor	Task sequence = “A sequence”	Mandate reassigned
Mandate submission	(1) To the task page, select the page to submit, (2) upload the resources to be submitted, click finish the task	Whether to expand personnel = existing personnel,” “task phase = ” model,” operating software = “Maya2016”, deadline = “2020-4-27”	Resource upload success, task status changed to completed pending review
Task sequence creation	(1) Into the task assignment module task sequence function, (2) click create task sequence, (3) fill in task sequence information and click finish	Task name = “A model”	Task sequence creation successfully

**Table 6:** Statistical analysis module test table.

Test function	Test operation	Test data	Test results
Project perspective statistical mission	(1) Go to the single task statistics page, click the project angle statistics tab page, (2) select the start date, select the end date, and select the project	Start date = 2019-05-01 end date = 2019-12-10 select item = “A”	Successfully display information on the number of tasks of A items in the selected time period
Statistical missions from a personnel perspective	(1) Go to the single task statistics page, click task angle statistics tab page, (2) select the start date, select the end date, and select the person who needs to count	Start date = “2019-05-01”, end date = “2019-12-10” personnel = “Zhang San”	Successful display of information on the number of tasks related to Zhang San in the selected time period
Task sequence-based statistics	(1) Go to the single task statistics page, click the task angle statistics tab page, (2) select the start date, select the end date, and select the required statistics task sequences	Start date = “2019-05-01” end date = “2019-12-10” select task sequence = “A sequence”	Accurate display of the number of tasks contained in the selected sequence of tasks within the selected time period
Project perspective statistical task sequence	(1) Selection of tasks under statistical analysis sequence statistics function, enter about statistics of the task sequence tab page, (2) select the start and end times, and select the animation project	Project = ” A “start date = “2019-05-01 end date “2019-12-10”	Accurate display of all task sequences under the project at different times

## Data Availability

The data used to support the findings of this study are available from the corresponding author upon request.
